# Pyrolytic Characteristics and Kinetics of *Phragmites australis*

**DOI:** 10.1155/2011/408973

**Published:** 2011-10-04

**Authors:** Hui Zhao, Huaxiao Yan, Congwang Zhang, Xiaodong Liu, Yanhui Xue, Yingyun Qiao, Yuanyu Tian, Song Qin

**Affiliations:** ^1^College of Chemical and Environmental Engineering, Shandong University of Science and Technology, Qingdao, Shandong 266510, China; ^2^Institute of Oceanology, Chinese Academy of Sciences, Qingdao, Shandong 266071, China; ^3^Graduate University, Chinese Academy of Sciences, Beijing 100049, China; ^4^Shandong Provincial Key Laboratory of Low-Carbon Energy Chemical Engineering, Qingdao 266510, China; ^5^Yantai Institute of Coastal Zone Research, Chinese Academy of Sciences, Yantai, Shandong 264003, China

## Abstract

The pyrolytic kinetics of *Phragmites australis* was investigated using thermogravimetric analysis (TGA) method with linear temperature programming process under an inert atmosphere. Kinetic expressions for the degradation rate in devolatilization and combustion steps have been obtained for *P. australis* with Dollimore method. The values of apparent activation energy, the most probable mechanism functions, and the corresponding preexponential factor were determined. The results show that the model agrees well with the experimental data and provide useful information for the design of pyrolytic processing system using *P. australis* as feedstock to produce biofuel.

## 1. Introduction

The common reed, *Phragmites australis* (Cav.) Trin. exSteud., described as one of the most widely distributed angiosperms [1, 2] is commonly found in freshwater and marine wetlands and along the upland edge of tidal marshes. *P. australis* has increased in proportion in both tidal and nontidal wetlands in North America and has become a major concern to wetland ecologists [3, 4]. Expansion of *P. australis* into salt marshes reportedly caused a fivefold decrease in plant species richness [5], reductions in microtopography [6, 7], and reductions in biodiversity [3]. *P. australis* is actively colonizing wetlands raising concern about loss of biodiversity [8, 9]. In addition, the alternative source of energy contributes to the reduction of CO_2_ emissions since the same amount of CO_2_ is extracted from the air during the growth period of the plants [10]. *P. australis* seems to be especially a promising energy plant and chemical feedstock due to its high production potential. Various processes have been proposed for the integrated utilization of these powerful plants [11, 12]. The common reed can provide a large quantity of biomass and annual yields of 40–63 tons per hectare have been reported. *P. australis* is becoming very important as stable biomass source for China with its natural huge reserves and increasing artificial cultivation.

Pyrolysis is an effective method to harvest the energy in *P. australis*. However, pyrolytic characteristics studies of *P. australis* are little, which has led to the production of biofuel being far from commercialization. Both the development of the pyrolytic process and reactor design require complete elucidation of the pyrolytic mechanism. Therefore, the pyrolytic mechanisms of *P. australis* should be studied.

The specific objective of the present work was to study the pyrolytic characteristics of *P. australis* using a TG/DSC instrument. The kinetic parameters of decomposition were then obtained and the pyrolytic mechanism was illustrated, which can provide useful information for the design of pyrolysis system using *P. australis *biomass as feedstock to produce bio-fuel.

## 2. Materials and Methods

### 2.1. Materials

*P. australis *samples were harvested from a wetland beside the campus of Shandong University of Science and Technology, Qingdao, China, September 2010.

### 2.2. Analysis of *P. australis* Samples

Samples were ground into fine powders and sieved to less than 0.147 mm. Proximate and ultimate analyses were carried out to characterize *P. australis* samples according to national standard GB212-91 (China) and element analyzer (Elementar Analysensysteme GmbH vario EL cube), respectively. In addition, cellulose, hemicelluloses, and lignin contents were analyzed according to methods from the literature [13]. The results were summarized in Table 1. All tests were carried out in triplicate.

### 2.3. Pyrolysis of the Samples

Thermogravimetric analyses were carried out using a thermal analyzer (TGA/DSC1/1600LF, METTLER TOLEDO Co., Switzerland). 10 mg of initial sample was pyrolyzed under a nitrogen flow rate of 50 cm^3^/min with a heating rate of 25°C/min. The weight loss and calorific changes in response to temperature were then recorded and used to plot the thermogravimetric analysis (TGA), derivative thermogravimetric analysis (DTG), and differential scanning calorimetric (DSC) curves. All experiments were replicated three times.

### 2.4. The Kinetic Parameters of the Samples

(1) $$\frac{d\alpha }{dt}=\kappa f\left(\alpha \right),$$

where **α** is the conversion rate and is defined as

(2) $$\alpha =\frac{m_{0}-m}{m_{0}-m_{\infty }},$$

*κ* is the velocity constant, and *f*(*α*) is the mechanism function with different relative coefficient

(3) $$T=T_{0}+\beta t,$$

where **β** is the heating rate and* T_0_* is the initial temperature.

The reaction rate constant, *κ*, can be described by the following expression:

(4) $$\kappa =CT^{m}.$$

By separation of variables and integration,

(5) $$\begin{array}{cc} G\left(\alpha \right) & =\overset{\alpha }{\underset{0}{\int }}\frac{d\alpha }{f\left(\alpha \right)}=\frac{C}{\beta \int_{T_{0}}^{T}T^{m}dT}=\frac{C}{\beta \left(m+1\right)}T^{m+1}+D\\ & =BT^{m+1}+D\overset{D=0}{⇄}BT^{m+1}. \end{array}$$

The expression of *G*(*α*) corresponding to each one of the mechanisms considered is also shown in Table 2

(6) $$\text{lg}G\left(\alpha_{i}\right)=\text{lg}B+\left(m+1\right)\text{lg}T_{i}.$$

This equation is used to estimate the most correct reaction mechanism function *G*(*α*). According to the plotting lg*G*(*αi*) versus lg*Ti* and a linear regression, if the mechanism studied conforms to certain *G*(*α*) function, the slope of the straight line should be equal to −1.00000 and the linear correlation coefficient *R^2^* is high

(7) $$B=\frac{C}{\beta \left(m+1\right)}.$$

Both of (5) and (6) are called Harcourt-Fission model of integral. *m* and *B* were constructed with deducing coefficients of least-square method

(8) $$\text{lg}\kappa_{i}=\text{lg}C+m\text{lg}T_{i}.$$

With a given value of lg*Ti*, the constants *C*, *m*, lg*κ*_*i*_  can be determined

(9) $$\ln\;\kappa_{i}=\ln\;A-\frac{E}{RT_{i}}.$$

Substituting the value of ln *κ*_*i*_ back into (9) in conjunction with 1/*T* allows ln*A* and *E* to be calculated [14].

All plots were generated and the lines were fitted using the Origin 8.0 software.

## 3. Results and Discussion

### 3.1. Characterization of Materials

The results of proximate, ultimate, and component analysis of *P. australis* samples are summarized in Table 1, which is in the same order of magnitude as energy crops. The comparison with other terrestrial materials shows a higher amount of ash and cellulose. The volatile matter and lignin contents of *P. australis* are lower, respectively, while the sulfur content of the samples is approximately equal [15].

### 3.2. Characteristics of the Thermal Degradation Process

Three stages in the pyrolytic process of *P. australis* are in accordance with the conclusion of oxidative pyrolysis curves of energy crops followed the usual shape for lignocellulosic materials [16–19]. The first stage (I) occurred as the temperature increased from 50 to 240°C, losing cellular water and the external water bound by surface tension. While the second stage (II), occurring as the temperature increased from 240 to 500°C was the devolatilization stage, during which the main pyrolytic process occurred and most of the organic materials are decomposed accompanied by various volatile components released gradually, resulting in a large weight loss which is more than 50% of total volatiles and formation of the main pyrolytic production. Specifically, stage II was composed of two zones for *P. australis* due to the different thermal stability of the components with zone I occurring as the temperature increased from 240 to 318°C which is a strong peak in the rate of weight loss curve, at which the rate of weight loss attains maximum with a maximum weight loss point at 288°C and zone II occurred as the temperature increased from 318 to 500°C with a maximum weight loss point at 346°C. The third stage (III) occurred as the temperature increased from 500 to 800°C with the residuals, the carbonaceous matters in the solid, slowly decomposed, resulting in the formation of a loose porous residual. A slight continued loss of weight is shown in the weight loss curve. *P. australis* is composed of many polysaccharides that have low polymerization. Moreover, the inorganic salts in *P. australis* presented a catalytic effect [20]. These findings indicate that the beginning of the decomposition occurs at a higher temperature for the samples evaluated in this study than for other terrestrial biomass with a high content of cellulose (straws and grasses) or lignin (woody biomass).

**Figure 1:**
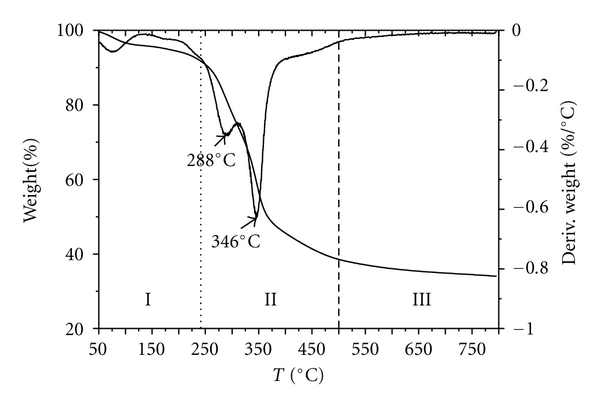
TG-DTG curves of *P. australis*.

**Figure 2:**
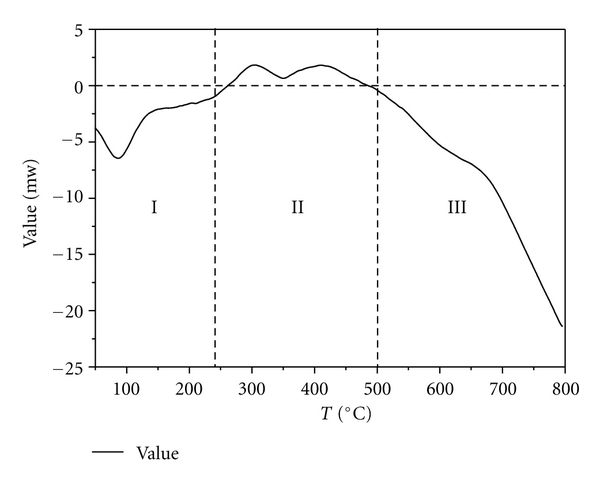
DSC curves of **P. australis*. *

**Figure 3:**
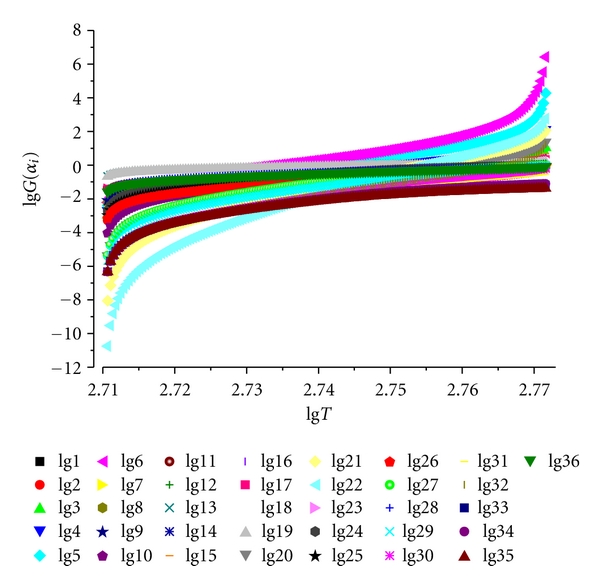
Plot for determination of most probable mechanism functions.

The weight losses of *P. australis* during stage I are primarily due to the loss of moisture and are similar to the moisture content values reported in Table 1. The instantaneous maximum reaction rate occurred in zone I of stage II. There was an endothermic peak during stage I that corresponded with the moisture evaporation procedure. As the temperature increased, an exothermic effect appeared during stage II and exothermic peaks were observed at 5–15°C after the maximum weight loss point. These findings indicate that the devolatilization stage (stage II) produced heat. Specifically, there was an endothermic effect during stage III. These findings indicate that the carbonaceous residual may have been decomposed at temperatures above 600°C. A maximum exothermic peak corresponding to the maximum weight loss rate peak appeared on the DSC curve of the samples (stage II). These findings indicate that the main pyrolysis process of the samples is an exothermic process. The exothermic effect was due to the charring process, which is the decomposition of the inorganic materials to metal carbonate (Figures 1, 2, and 3) [21, 22]. 

### 3.3. Kinetic Analysis of the Pyrolysis Process

The most probable mechanism function with integral form can be expressed by  *G*(*α*_3_) = (1−*α*)^1/2^ − 1,

(10) $$\begin{array}{cc} m+1 & =\frac{n\sum_{i=1}^{n}\text{lg}G\left(\alpha_{i}\right)\text{lg}T_{i}-\sum_{i=1}^{n}\text{lg}T_{i}\sum_{i=1}^{n}\text{lg}G\left(\alpha_{i}\right)}{n\sum_{i=1}^{n}{T_{i}}^{2}-\sum_{i=1}^{n}T_{i}\sum_{i=1}^{n}T_{i}},\\ \text{lg}B & =\frac{\sum_{i=1}^{n}T_{i}G\left(\alpha_{i}\right)\sum_{i=1}^{n}T_{i}-\sum_{i=1}^{n}G\left(\alpha i\right)\sum_{i=1}^{n}{T_{i}}^{2}}{\sum_{i=1}^{n}T_{i}\sum_{i=1}^{n}T_{i}-n\sum_{i=1}^{n}{T_{i}}^{2}}, \end{array}$$

and the results calculated according to the above equations are as follows: lg*B* = −116.34877, *m* = 41.18814, and lg*C* = −113.32564.

**Figure 4:**
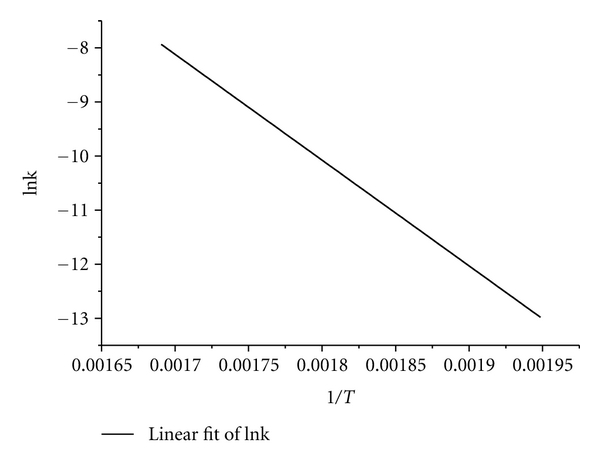
Plot for determination of *E* and *A*(ln *A*  = 25.14 min^−1^, *E *= 162.66 kJ mol^−1^, *R^2^*  = 0.9999).

The result indicates that the model is in good agreement with the experimental data (Figure 4).

Comparisons of the decomposition temperature and activation energy of several types of biomass are provided in Table 3 [17]. The results indicate that the decomposition temperature of *P. australis* is lower than that of several kinds of plants and single component of biomass. 

## 4. Conclusions

High priority should be given to the development and protection of biomass pyrolysis which is widely recognized as a technically and economically feasible way. This study presents useful information for the design of a pyrolytic processing system using *P. australis *biomass.

- There were three stages in the pyrolytic process of the samples. The second stage is the main pyrolysis process and most of the organic materials are decomposed in this stage which is our study focused on.
- Iterative isoconversional procedure has been applied to estimate the values of apparent activation energy. The method of Dollimore combined with 36 mechanism functions is used to define the most probable mechanism *G*(*α*_3_) = (1 − *α*)^−1/2^ − 1; the preexponential factor *A *= 8.28 × 10^10^ min^−1^ and *E *= 1.63 × 10^5^ J mol^−1^ are obtained on the basis of *G*(*α*).
- Comparisons of various kinetic parameters of pyrolysis for different biomass types show that* P. australis *biomass has a great potential and a good prospect for producing biofuel by fast pyrolysis process.

To learn more about *P. australis* pyrolysis, characterization of liquid and solid products should be more in-depth understood [26, 27].

## Figures and Tables

**Table 1:** Proximate analysis, ultimate analysis, and component analysis of *P. australis* (/%wt).

Parameter	* P. australis*
*Proximate analysis * ^ a^ (wt.%, ad. Basis)	
Moisture	5.89 ± 0.03
Ash	12.32 ± 1.12
Volatile matter	70.01 ± 2.40
Fix carbon^c^	11.78
*Ultimate analysis * ^ b^ (wt.%, daf. Basis)	
Carbon	42.78 ± 1.53
Hydrogen	5.17 ± 0.02
Oxygen^c^	50.511
Nitrogen	1.31 ± 0.02
Sulfur	0.229 ± 0.01
C/H	8.31 ± 0.31
C/N	32.57 ± 1.55
*Component analysis *(wt.%, daf. Basis)	
Hemicellulose	30.68 ± 3.15
Lignin	20.34 ± 1.56
Cellulose	43.05 ± 3.98

^a^Dry-free basis; ^b^Dry ash-free basis;  ^c^Calculated by difference.

**Table 2:** Algebraic expressions of functions *G*(*α*) and *f*(*α*) and mechanisms [23, 24].

No.	*G*(*a*)	*f*(*α*)	Rate-determining mechanism
1	1 − (1−*α*)^2/3^	3/2(1−*α*)^1/3^	Chemical reaction
2	1 − (1−*α*)^1/4^	4(1−*α*)^3/4^	Chemical reaction
3	(1−*α*)^−1/2^ − 1	2(1−*α*)^3/2^	Chemical reaction
4	(1−*α*)^−1^ − 1	(1−*α*)^2^	Chemical reaction
5	(1−*α*)^2^ − 1	1/2(1−*α*)^3^	Chemical reaction
6	(1−*α*)^3^ − 1	1/3(1−*α*)^4^	Chemical reaction
7	1 − (1−*α*)^2^	1/2(1 − *α*)	Chemical reaction
8	1 − (1−*α*)^3^	1/3(1−*α*)^2^	Chemical reaction
9	1 − (1−*α*)^4^	1/4(1−*α*)^3^	Chemical reaction
10	*α* ^3/2^	2/3*α* ^−1/2^	Nucleation
11	*α* ^1/2^	2*α* ^1/2^	Nucleation
12	*α* ^1/3^	3*α* ^2/3^	Nucleation
13	*α* ^1/4^	4*α* ^3/4^	Nucleation
14	*ln* *a*	*α*	Nucleation
15	−*ln*(1 − *α*)	1 − *α*	Assumed random nucleation and its subsequent growth
16	[−*ln*(1−*α*)]^2/3^	3/2(1 − *α*)[−*ln*(1−*α*)]^1/3^	Assumed random nucleation and its subsequent growth
17	[−*ln*(1−*α*)]^1/2^	2(1 − *α*)[−*ln*(1−*α*)]^1/2^	Assumed random nucleation and its subsequent growth
18	[−*ln*(1−*α*)]^1/3^	3(1 − *α*)[−*ln*(1−*α*)]^2/3^	Assumed random nucleation and its subsequent growth
19	[−*ln*(1−*α*)]^1/4^	4(1 − *α*)[−*ln*(1−*α*)]^3/4^	Assumed random nucleation and its subsequent growth
20	[−*ln*(1−*α*)]^2^	1/2(1 − *α*)[−*ln*(1−*α*)]^−1^	Assumed random nucleation and its subsequent growth
21	[−*ln*(1−*α*)]^3^	1/3(1 − *α*)[−*ln*(1−*α*)]^−2^	Assumed random nucleation and its subsequent growth
22	[−*ln*(1−*α*)]^4^	1/4(1 − *α*)[−*ln*(1−*α*)]^−3^	Assumed random nucleation and its subsequent growth
23	*ln* *a*/(1 − *α*)	*a*/(1 − *a*)	Branching nuclei
24	*α*	(1−*a*)^0^	Contracting disk
25	1 − (1−*α*)^1/2^	2(1−*a*)^1/2^	Contracting cylinder (cylindrical symmetry)
26	1 − (1−*α*)^1/3^	3(1−*a*)^2/3^	Contracting sphere (spherical symmetry)
27	*α* ^2^	1/(2*a*)	One-dimensional diffusion
28	[1−(1−*α*)^1/2^]^1/2^	4{(1−*α*)[1− (1−*α*)]^1/2^}^1/2^	Two-dimensional diffusion
29	*a* + (1 − *α*)*ln*(1 − *α*)	[−*ln*(1−*α*)]^−1^	Two-dimensional diffusion
30	[1− (1−*α*)^1/3^]^2^	(3/2)(1−*α*)^2/3^[1− (1−*α*)^1/3^]^−1^	Three-dimensional diffusion, spherical symmetry
31	1 − 2/3*α* − (1−*α*)^2/3^	(3/2)[(1−*αa*)^−1/3^−1]^−1^	Three-dimensional diffusion, cylindrical symmetry
32	[(1−*α*)^ −1/3^ −1]^2^	(3/2)(1−*α*)^4/3^[(1−*α*)^−1/3^−1]^−1^	Three-dimensional diffusion
33	[(1+*α*)^1/3^ −1]^2^	(3/2)(1+*α*)^2/3^[(1+*α*)^1/3^−1]^−1^	Three-dimensional diffusion
34	1 + 2/3*α* − (1+ *α*)^2/3^	(3/2)[(1+*α*)^−1/3^−1]^−1^	Three-dimensional diffusion
35	[(1+*α*)^−1/3^−1]^2^	(3/2)(1 + *α*)4/3[(1 +*α*)^−1/3^−1]^−1^	Three-dimensional diffusion
36	[1− (1−*α*)^1/3^]^1/2^	6(1 − *α*)2/3[1− (1−*α*)^1/3^]^1/2^	Three-dimensional diffusion

**Table 3:** Comparison of various kinetic parameters of pyrolysis for different biomass[17, 25].

Samples	Decomposition temperature (°C)	Activation energy (kJ mol^−1^)
Cellulose	300–430	200
Hemicellulose	250–350	100
Lignin	250–550	80
Chitosan	268–312	95.6–185.7
*Enteromorpha prolifera*	174–551	228.1
*spirulina platensis*	190–560	76.2–97
*Chlorella protothecoides*	150–540	42.2–52.5
*Dunaliella tertiolecta*	155–299	145.713–146.421
Oak tree	230–400	236.2
Corn stover	250–470	57.95–58.94
Peat	208–334	52.77
*P. australis*	240–500	163
